# “…they think we are conversing, so we don’t care about them…” Examining the causes of workplace violence against nurses in Ghana

**DOI:** 10.1186/s12912-016-0189-8

**Published:** 2016-11-28

**Authors:** Isaac Mensah Boafo

**Affiliations:** Department of Sociology, University of Ghana, Legon, Accra, Ghana

## Abstract

**Background:**

This study is part of a larger project aimed at exploring the workplace experiences of nurses working in public general hospitals in Ghana. The current paper explores the causes of workplace violence against nurses in Ghana.

**Methods:**

Twenty-four semi-structured in-depth interviews were conducted with professional nurses working in five regions of Ghana. They were selected through purposive and participant-to-participant snowball sampling techniques. Data was analysed through thematic analyses.

**Results:**

The findings of the study suggest that nurses are not (always) passive recipients of violence. Workplace violence can be instigated by either of the parties to the nurse-patient/relative interaction. Nurses’ accounts of the causes of violence suggest that violence could be instrumental or reactive. The study further suggests that the causes of violence may differ depending on which party instigated the violence. The main causes of violence identified include ineffective communication, long waiting times and perceived unresponsiveness, and enforcement of visiting hours.

**Conclusion:**

It is concluded that workplace violence could be reduced through the provision of adequate information to patients and their relatives. Nurses could also be trained in effective communication and interpersonal skills; and on how to identify and avoid potentially violent situations. It is also imperative that policies and measures aimed at addressing workplace violence are instituted to address the problem. Mass education may also be carried out to sensitise the general public on the adverse effects of violence against nurses.

## Background

### Introduction

This paper is the third in a series of six papers aimed at exploring workplace experiences of nurses working in public general hospitals in Ghana. The aim of the current paper is to explore the main causes of workplace physical and verbal abuse against nurses by patients and relatives of patients from nurses’ perspective. In two earlier papers, it was reported that violence is a major problem for nurses in Ghana. Incidence rates of 9%, 12.2% and 52.7% were reported for physical violence, sexual harassment and verbal abuse [5]. Although many studies have been conducted on workplace violence against nurses, most of these studies used quantitative methods to establish the incidence, sources and effects of violence on nurses [1, 13, 14, 17, 19, 27]. Such studies, though very valuable, tend to present nurses as passive recipients of workplace violence. This paper argues that violence could be instigated by any of the parties to the nurse-patient/relative interaction.

The causes of workplace violence established in the literature include long waiting times, unmet treatment outcomes, mental illness, use of alcohol and other illicit drugs, access to weapons and the physical hospital environment in general [8, 16, 17, 29]. However, the main causes of violence may differ from one context to another based on differences in socio-cultural setting, the organisation of healthcare, staff training, etc. It is therefore important that the causes of violence pertaining to a particular socio-cultural milieu are identified to enable context-specific measures to be taken to address the problem. Indeed studies have shown that measures taken by countries such as Australia to ensure the safety of its nurses have not been effective due to lack of in-depth investigation into the phenomenon [9].

The causes of violence identified in the current paper include long waiting times, perceived unresponsiveness, enforcement of visiting hours, ineffective communication and the use of harsh (abusive) language by some nurses. Patients and their relatives resort to violence when they perceive they are not being given prompt attention. This use of violence in such instance is thus a means to achieve some outcome. Violence from patients and their relatives may also be an emotional response to rude treatment (abuse) from nurses. It is, therefore, argued that the use of violence by patients and their relatives could be instrumental, reactive or both. Violence could be used as a means of obtaining justice or some desired outcome or as a reaction to the attitudes and behaviours of nurses.

### Literature review

Workplace violence is a growing social phenomenon which has caught the attention of researchers and policy makers in many countries worldwide. It is a multi-causal and multi-faceted problem [3]. It refers to ‘incidents where staff are abused, threatened or assaulted in circumstances related to their work, including commuting to and from work, involving an explicit or implicit challenge to their safety, well-being or health’ ([15], 2). Violence is a generic term that covers all kinds of abuse, which humiliates, degrades or damages a person’s well-being, dignity and value. Physical violence refers to “the use of physical force against another person or group, that results in physical, sexual or psychological harm” ([15], 3). Physical violence includes beating, kicking, slapping, stabbing, shooting, pushing, biting, pinching and grabbing. Verbal violence is defined as any communication that attacks a person professionally or personally; it may refer to behaviours such as yelling, verbal insults, or threats of harm [15].

Healthcare workers, particularly nurses, are at high risk of workplace violence as the nursing profession is increasingly becoming associated with workplace violence, [1, 13, 14, 17, 19, 27]. Besides its direct effects on victims, workplace violence against nurses affects the healthcare system in general as it increases turnover rates. It also reduces the quality of care [5, 26]. The most frequently reported perpetrators of violence are patients and their relatives.

Although the negative psychological and physical effects of workplace violence against nurses are firmly established in the literature [5, 12, 18, 22, 26], its causes have been underexplored. This is largely due to the quantitative nature of most of the studies on workplace violence against nurses. Recent studies have largely been interested in presenting the incidence rates and effects of violence against nurses (see for instance, [1, 13, 14, 17, 19, 27]). In doing so, nurses have often been presented as passive recipients of workplace violence. The part played by nurses in instigating violence against them is often missed by these studies. However, a few studies have suggested that long waiting times, use of alcohol and illicit drugs, access to guns and other weapons, attitudes and behaviours of nursing staff, and unexpected treatment outcomes among others are major precipitators of violence against nurses [8, 16, 17, 29]. The aim of this study is to examine the causes of violence against nurses within the Ghanaian setting. As will be shown later in this paper, workplace violence is a two-way affair, instigated by both nurses and patients and their relatives.

### Theoretical perspective

This study is informed by the symbolic interactionist perspective. This perspective is at the centre of sociological analyses of social interaction at the micro-level. According to the interactionist perspective, meanings are created through social interaction and modified through interpretation [20]. People interact according to how they perceive a situation; how they understand the social encounter; and the meanings they bring to it [21]. This perspective sees interpersonal and situational factors as important in understanding workplace violence [23]. It recognizes that perpetrators “often view their own behaviour as legitimate and even moralistic. Thus beliefs about justice and equity, the assignment of blame, and the accounts that people give to excuse or justify their behaviour are central” ([11], 2). Violence can be divided into two broad categories, namely, instrumental and reactive [23]. The former refers to the situation where the violent act is used to obtain some valued outcome, and the latter is an emotional response or reaction to a situation. Instrumental violence is thus a means to an end. In their ground-breaking work on the interactionist approach to violence, Felson and Tedeschi [11] contended that instrumental violence is employed to influence or coerce others, to establish and protect valued social identities or to attain justice or retribution. Reactive violence is impulsive, thoughtless, driven by anger and aimed at harming the victim. It is generally seen as a reaction to some perceived provocation. However, this distinction between purely instrumental and reactive violence is not always possible to make as a single act of violence may be triggered by mixed motives [11]. Applying this perspective to the current study, it is argued that violence is used by patients and thier relatives to obtain justice or certain desired outcomes or as a reaction to the behaviour of nurses.

## Methods

### Participants

The study reported in this paper is part of a larger study aimed at investigating the challenges and problems facing nurses in Ghana. The original study involved a mixed methods research design. It included six key informant interviews, 24 semi-structured in-depth interviews and 592 cross-sectional questionnaire surveys. Participants were drawn from two teaching hospitals, five regional and five district hospitals in Ghana. In all, there were three teaching hospitals, nine regional hospitals and over a 100 district hospitals in Ghana.

To ensure that the Northern and Southern divide of the country are represented, the Tamale and Korle Bu Teaching Hospitals were included in the design. Five out of the ten administrative regions of the country were randomly selected for the study - Greater Accra, Eastern, Volta, Ashanti and Northern Regions. From each of these regions, the regional hospital was chosen and a district hospital was randomly chosen from that region. This yielded five district hospitals and five regional hospitals. All of the 10 regions had a regional hospital with the exception of the Ashanti Region where no hospital was designated as such by the Ghana Health Service. In view of this, a government hospital located in Kumasi (the capital of the Ashanti Region) was chosen to represent a regional hospital for the purposes of this study. For the purposes of the current paper, the qualitative procedures involving the 24 semi-structured in-depth interviews are reported. The six key informants were removed from the analyses as they were not asked about the causes of workplace violence, which is the focus of this paper.

Permission letters were written to the various health facilities selected for the study. After permission had been obtained, initial meetings were arranged with the directors of nursing services in the various hospitals, where the study was explained to them. After each of these meetings, the researcher made contacts with nurses in the hospital and those who were deemed to be resourceful for the purposes of the study, were requested to take part in the qualitative interviews [7]. These selections were made with the help of the heads of nursing services and through the snowballing technique. The researcher was introduced to one or two nurses by the heads of nursing services, and those who agreed to participate in the study were interviewed. Interviewees were also asked to suggest other eligible nurses for participation in the study. This snowballing process was continued until data saturation was reached.

Interviews were conducted at a time and place convenient for the participants, and safe for both the participant and the researcher. This ensured minimal disruption in the activities of the participants. Most of the interviews were conducted in empty offices or spaces in the hospital where the confidentiality of the participants could be ensured, and had low noise levels. Two interviews were conducted in the office of the researcher in Accra and two others were conducted over telephone. One of the interviews was conducted in the house of the interviewee. All interviews were conducted by the researcher. All interviews were conducted in English, and were audio-recorded with the consent of the interviewees.

Member checking was employed to ensure the accuracy and authenticity of the qualitative data. Authenticity here refers to presenting a fair, honest, and balanced account of social life from the point of view of the participants [24]. In this study, interview transcripts were taken to some of the interviewees to check the accuracy of the data. In all cases, no changes were suggested by participants. Other participants were too busy with work and other personal duties and could not go through their interview transcript. This setback was however offset by the fact that throughout the interview process, the researcher summarized the information provided and questioned interviewees for confirmation and clarifications.

### Data organisation and analysis

Thematic analysis was employed in analysing the data. The analyses were guided by the steps suggested by Braun and Clarke [6]. Since the data was collected by the researcher, the analysis was approached with some prior knowledge and analytical interpretations. The data analysis began with the transcription of the interviews. The transcription of the interviews was completed by the researcher and one research assistant. All interviews were transcribed verbatim. The interview transcripts were then uploaded into the qualitative data analysis software, NVivo (Version 10). The interview transcripts were then read over and over and where necessary, the audio recordings of the transcripts were played to rectify errors in the transcripts. Through this process of reading and re-reading, researcher became familiar with all aspects of the data, and ideas for coding were jotted down. After this ‘familiarization tour’ of the data, actual coding of the data began using NVivo. As far as possible, every data item was given equal attention, and as many codes as possible were created. The various codes were placed under appropriate potential themes in the form of ‘parent nodes’ in NVivo. The development of the themes was theory driven [6] as they were created with specific research question in mind.

The extracts under each code and theme were read to ensure that they fit into where they have been placed. The themes themselves were refined - where necessary themes were modified or changed to reflect the codes they contain and to ensure there is not too much overlap between themes. Some of the codes became themes and others became sub-themes. After this exercise, the data was searched again with the aim of coding any additional data that was missed during the coding process. Codes which were found not to fall under any of the themes of interest were put aside. The themes that remained are those that tell a story about the data in relation to the research question. The analyses mainly involved searching through the entire data set to identify repeated patterns of meaning. The findings presented in the current paper relates to the causes of workplace physical and verbal abuse against nurses. All extracts presented in the current paper were produced verbatim.

## Results and discussion

### Demographic characteristics

The data presented in this paper involved 24 professional nurses. In terms of gender composition, 9 were males and 15 were females. Regarding the positions they occupied, one was a Deputy Director of Nursing Services (DDNS); four were Principal Nursing Officers (PNO); three were Senior Nursing Officers (SNO); five were Nursing Officers (NO); four were Senior Staff Nurses (SSN); and six were Staff Nurses (SN). The majority (12) had Diploma qualifications, and about one-third (9) were within the age group of 21–30 years. In terms of the type of hospitals in which they worked, nine worked in teaching hospitals, eleven worked in regional hospitals and four worked in district hospitals. The socio-demographic characteristics of the participants in the qualitative component of this research are displayed in Table 1.

**Table 1:** Socio-demographic Characteristics of Participants (*N* = 24)

Item description	*n*
*Gender*	
Male	9
Female	15
*Age group*	
21–30	9
31–40	7
41–50	4
51–60	4
*Educational attainment*	
Certificate	4
Diploma	12
Degree	6
Advanced Degree	2
*Position*	
DDNS	1
PNO	5
SNO	3
NO	5
SSN	4
SN	6
*Type of Hospital*	
Teaching hospital	9
Regional hospital	10
District hospital	5

Source: Qualitative Interviews 2013–2014

### Causes of workplace violence

The causes of violence against nurses presented in this paper focus on the immediate hospital environment, and the meanings that are constructed during interactions with nurses. Erickson [10] suggested in her application of the interactionist perspective to intimate partner violence that, attention must be paid to the phenomenology of intimate violence. This is because violence is considered to be a “situated, interpersonal, emotional and cognitive activity involving negative symbolic interaction” (p. 529). Several causes of workplace violence against nurses have been documented in the literature, which suggest the need to focus on the immediate context within which violence occurs [29]. Felson and Tedeschi [11] contended in their seminal work on the interactionist approach on aggression that, violence can be used as a means to influence or coerce others; it can also be used to attain justice or retribution. The main causes of violence against nurses by patients and their relatives in the present study can be seen in this light. Nurses’ responses to the question of what usually triggers violence (verbal and physical) by patients and their relatives were categorised under waiting times and perceived unresponsiveness, perceived favouritism, visiting hours, and communication.

### Waiting time and perceived unresponsiveness

The length of waiting period, and the perception that nurses are unresponsive to the plight of patients was one of the main causes of violence against nurses. The data revealed that nurses at the outpatient department (OPD) were the worse victims as far as this cause of violence is concerned. Nurses at the OPD take the initial decision of which patient gets seen by which doctor; they categorise and process patients to be seen by specific doctors based on their initial assessment of the condition presented (triage system). After this assessment, patients still have to wait for long hours before a medical doctor sees them (due to inadequate number of medical doctors). In the course of waiting, they tend to believe that nurses are responsible for those delays, which sometimes leads to violence against the nurses. Abena (female SNO) expressed this opinion when she attributed violence from patients and their relatives to the fact that they are always in a hurry for treatment:

*…everybody is in a hurry but l don’t know where we are going. Sometimes patients insult us at the OPD because they are in a hurry and they think we are not being fast enough.*

This finding is consistent with much of the literature. For instance, a Nigerian study also found long waiting times to be associated with violence against healthcare professionals [3]. In an Australian study, Bakker [4] also suggested that patients and their relatives erroneously think nurses have power over waiting times. Patients and relatives, thus, get angry, swear and harass nurses when they have to wait a long time for their turn. However, unlike other studies [3, 29] alcohol intoxication and the use of illicit drugs did not emerge as major causes of violence in the current study.

Closely related to the issue of long waiting times is perceived unresponsiveness. Unresponsiveness refers to the failure to respond quickly to a person or an event. The data revealed that patients and their relatives in their anxious state want to see nurses working promptly and briskly to help them, especially when they perceive their case to be an emergency one. The data showed that where patients or their relatives feel that they are not getting the prompt care they need, it triggers violence against nurses who are the first point of contact. This could explain why several studies [25, 31] have reported that nurses at emergency and out-patient departments were more likely to suffer the most abuse from patients and their relatives. As reported in our previous paper [5], nurses at the OPD had the highest incidence rates of workplace verbal violence.

The data reported in the current paper suggested that nurses at the OPD were sometimes seen by patients and their relatives as idling around as they wait for doctors to request patients’ folders. Patients and their relatives thus see nurses at the OPD as not doing their job and for that matter being the cause of delays. The following quotations were typical of nurses’ views on how waiting times and its associated perceived unresponsiveness trigger abuse:

*Sometimes we don’t have enough doctors… so they keep long. And while we also sit and wait, we chat; you know we are human beings. In this situation, they [patients and their relatives] sit there and they think we are conversing, so we don’t care about them… when they see us talking then they start their insults (Efe, female SN).*

*…because they are sick, they think as soon as they come, we have to give them treatment. But you will come and meet someone else, so it’s a matter of them getting patient with us for us to render the service to them (Akoto, male SSN).*

The data suggested that this perception of unresponsiveness is the result of the lack of adequate healthcare personnel. Inadequate staffing translates into longer waiting times and a perception that healthcare professionals particularly nurses are the cause of this delays. Atswei, a nurse at one of the teaching hospitals who have practiced for more than 20 years re-echoed the shortage of staff as a cause of violence against nurses when she remarked;

*Have you been to the emergency room? It is really a busy place. You go there around 10am and you will see how the place is. Sometimes there are just two nurses checking round for blood sugar, BP, vitals etc. And can two nurses do all that? Several patients can come in at once referred from other hospitals. Even machines would break down but they want you to attend to them by force [they want to be attended to immediately] (Atswei, female PNO).*

The data revealed that the perception that nurses are not responsive to the plight of patients is actually a manifestation of the inadequate number of healthcare workers (doctors and nurses) available to deliver high quality and prompt health care to patients. As indicated earlier, the nurse-population and doctor-population ratios are very high compared to what pertains in other economically advanced countries [32]. Violence against nurses as a result of long waiting times and perceived unresponsiveness could thus be seen as an expression of dissatisfaction by patients and their relatives with care. Lack of insight into the roles of nurses may also contribute to this perception. However, this probable explanation was not evident in the data.

### Perceived favouritism

Perceived favouritism emerged as one of the main causes of violence particularly against nurses at the OPD. Patients attending the OPD at a hospital present different conditions with different levels of severity. Some patients may require immediate attention, as any further delay may result in complications or even death. In view of this, it becomes imperative that nurses prioritize which patient gets seen first based on the conditions presented and not based on the time of arrival at the hospital. The data revealed that violence is sometimes triggered by misconceptions of these prioritizations. Nurses related that, in their attempt to prioritise the conditions of patients so that those who need urgent attention could be attended to first, they get abused by other patients and their relatives as they think the *‘first-come-first-served’* principle must be followed.

*…sometimes too when you have a very bad case, you are not supposed to let the patient queue; they must be seen right way by a doctor. The patients tend to say that because you have taken bribe from the patient or because you know him or her, you have allowed her to cross the line then they would start insulting you (Ama, female NO).*

Although nurses spoke about violent episodes in times when they were trying to prioritize the health conditions of patients, it is also possible that favouritism actually occurs in these settings. Considering the socio-cultural context of Ghana, it is possible for nurses to allow their relatives and friends to jump queues irrespective of the severity of their conditions. Indeed an African study has found nepotism to be one of the causes of violence against nurses [30]. Indeed other studies have shown that violence is used by patients and their relatives to express dissatisfaction or to ensure their rights are respected. For instance, Al et al. [2] investigated the public’s view of violence towards healthcare professionals in Turkey. In that study, it was found that about 20% of the 1600 people sampled believed that violence is a method of claiming rights. And a Norwegian study found that 25% of primary health professionals (including nurses) who experienced verbal abuse and 3% of those who experienced physical violence were as a result of clients’ dissatisfaction with service.

### Enforcement of visiting hours

Regarding visiting hours, nurses reported that relatives of patients often abuse them because they have been refused entry into the wards after visiting hours. Relatives may sometimes be anxious to see their sick relations and may thus disregard visiting hours stipulated by the hospital. Others may simply be ignorant of the visiting hours. The data revealed that visiting hours is one of the major causes of workplace violence against Ghanaian nurses. All nurses interviewed on the various wards identified disagreement over visiting hours as a cause of workplace violence, particularly verbal abuse. Violence in this regard could be seen as being employed to achieve a desired outcome. The following excerpts illustrate this point:

*…we are always fighting with them [relatives of patients]. Anytime they come, they want you to allow them into the ward (Teiko, female SNO).*

*It wasn’t visiting hours but the relative wanted to enter, so I told him to wait a while because we are still doing consultation after which he can enter. He got pissed off and was saying all kinds of words. He said this place is not my father’s property so I cannot prevent him from entering into the ward. That is what some of us do and we don’t get anywhere. Those were the words he was using. He said so many things but I didn’t mind him (Issah, male SSN).*

Three nurses also linked violence resulting from denying relatives of patients (particularly males) entry into wards after visiting hours to gender constructions. They narrated that male relatives often feel offended when they are refused entry into wards by female nurses. It hurts their masculine ‘ego’ for which reason they resort to abusing these nurses. Atswei, a female PNO, illustrated this situation when she remarked:

*…a woman boss coming to tell me it’s not visiting time, who are you? And you know the problem? In the house, he is the boss and when they come to the ward a woman boss is coming to tell me you can’t enter the ward when it’s not visiting time. They [male relatives] get furious.*

### Interpersonal skills

Finally, and most importantly, communication issues were found to be a major contributing factor to workplace violence against nurses. The data suggested that the language (both verbal and non-verbal) used by some nurses in their interaction with patients and their relatives is an important factor as far as workplace violence is concerned. Nurses’ reports on the causes of workplace violence showed that the use of harsh and rude language by nurses is one of the major causes of violence against them by patients and their relatives. This finding is consistent with several other studies [8, 29, 30]. In this context, episodes of violence against nurses were reactions to the attitudes and behaviours of some nurses. They are responses elicited by the harsh or uncourteous language used by some nurses in their interaction with patients and their relatives.

*Sometimes the reaction of some of the nurses towards them [patients and their relatives] too you know makes them angry and agitated. Because of the anxiety and the frustration they go through, when they come [to the hospital], we need to talk to them nicely (Akweley, female SSN).*

Adwoa, a female PNO who has more than twenty years work experience put this more strongly when she stated, *“most nurses don’t have good attitude towards work… Most nurses are rude; it is not a perception (Adwoa, female PNO).* Adwoa’s comment suggests that, nurses’ style of communication with patients and their relatives could evoke violence against them. In a Chinese study, miscommunication was found to be the leading cause of violence against healthcare workers [8]. A participant in that study expressed it this way, “some medical staff don’t care about their patients and their detached facial expression and cold speeches led to complaints and abuses from patients and their relatives” ([8], 7). Patients and their relatives want to be listened to [2], they want to be shown compassion [28], and they are anxious for information on their condition and treatment [16]. Language, which tends to humiliate, fraught with apathy and rudeness and disrespect is likely to provoke violent behaviour [29, 30]. The findings of this study, thus makes it imperative that nurses are given regular refresher courses on communication and interpersonal skills.

## Conclusion

All the causes of verbal and physical workplace violence identified in the current paper, i.e. long waiting times and perceived unresponsiveness, perceived favouritism and enforcement of visiting hours could be reduced through good interpersonal and communication skills. If patients and their relatives are provided with adequate information on how long they have to wait, and why, and are made to understand that people with more serious conditions will be attended to in a more prompt manner irrespective of time of arrival, the perception of unresponsiveness and favouritism could be reduced. Applying the classification of Felson and Tedeschi [11], the causes of violence as identified by nurses themselves can be seen as instrumental, reactive (emotive) or both. It is a means through which patients and their relatives establish their dissatisfaction with, or ensure ‘fairness’ in service delivery. However, the use of violence cannot be an acceptable means to achieve desired outcomes. Patients and their relatives, and the general public must be educated and encouraged to channel their complaints to the appropriate hospital authorities. Based on the data presented in the current paper, it is recommended that patients and their relatives should be provided with adequate information on waiting times and hospital procedures and processes. Nurses could also be equipped with good communication and interpersonal skills through regular training so as to enhance the quality of their interactions with patients and their relatives.

## Abbreviations

DDNS: Deputy Director of Nursing Services
NO: Nursing Officer
OPD: Outpatient Department
PNO: Principal Nursing Officer
SN: Staff Nurse
SNO: Senior Nursing Officer
SSN: Senior Staff Nurse
